# Proteomic analysis of the phycobiliprotein antenna of the cryptophyte alga *Guillardia theta* cultured under different light intensities

**DOI:** 10.1007/s11120-017-0400-0

**Published:** 2017-05-24

**Authors:** Thomas Kieselbach, Otilia Cheregi, Beverley R. Green, Christiane Funk

**Affiliations:** 1 Department of Chemistry, Umeå University, 90187 Umeå, Sweden; 2 Botany Department, University of British Columbia, Vancouver, BC V6T 1Z4 Canada

**Keywords:** Cryptophyta, Phycobilin, Phycobiliprotein, Translation, TAT-pathway, Proteomics

## Abstract

Plants and algae have developed various light-harvesting mechanisms for optimal delivery of excitation energy to the photosystems. Cryptophyte algae have evolved a novel soluble light-harvesting antenna utilizing phycobilin pigments to complement the membrane-intrinsic Chl *a*/*c*-binding LHC antenna. This new antenna consists of the plastid-encoded β-subunit, a relic of the ancestral phycobilisome, and a novel nuclear-encoded α-subunit unique to cryptophytes. Together, these proteins form the active α_1_β·α_2_β-tetramer. In all cryptophyte algae investigated so far, the α-subunits have duplicated and diversified into a large gene family. Although there is transcriptional evidence for expression of all these genes, the X-ray structures determined to date suggest that only two of the α-subunit genes might be significantly expressed at the protein level. Using proteomics, we show that in phycoerythrin 545 (PE545) of *Guillardia theta*, the only cryptophyte with a sequenced genome, all 20 α-subunits are expressed when the algae grow under white light. The expression level of each protein depends on the intensity of the growth light, but there is no evidence for a specific light-dependent regulation of individual members of the α-subunit family under the growth conditions applied. GtcpeA10 seems to be a special member of the α-subunit family, because it consists of two similar N- and C-terminal domains, which likely are the result of a partial tandem gene duplication. The proteomics data of this study have been deposited to the ProteomeXchange Consortium and have the dataset identifiers PXD006301 and 10.6019/PXD006301.

## Introduction

Photosynthesis is the process that powers all life on Earth, generates renewable energy and food, and counteracts the greenhouse effect. Algae and cyanobacteria are without doubt the most productive photosynthetic organisms on Earth. The first step of photosynthesis is harvesting of sunlight by designated pigment-binding antenna complexes, which in the photosynthetic light reaction rapidly transfer the absorbed light energy to a reaction center (Blankenship 2014; Mirkovic et al. 2016). While the reaction centers of Photosystem II (PSII) and Photosystem I (PSI) remained highly conserved during evolution, various antenna systems have evolved in photosynthetic organisms. Prokaryotic cyanobacteria and eukaryotic red algae contain phycobilisomes as their major antennae-rods of stacked phycobiliproteins, to which the linear tetrapyrrole phycobilin pigments are covalently bound (Adir 2005; Watanabe and Ikeuchi 2013). These structures are extrinsically associated with the stromal side of the thylakoid membrane. In higher plants and green algae, the most abundant antenna is the chlorophyll *a*/*b*-binding light-harvesting complex (referred to as LHC), which is inserted into the thylakoid membrane (Neilson and Durnford 2009). As well as the phycobilisome, red algae also have a related LHC antenna, which binds only Chl *a* and is mainly associated with PSI (Gantt et al. 2003).

**Fig. 1:**
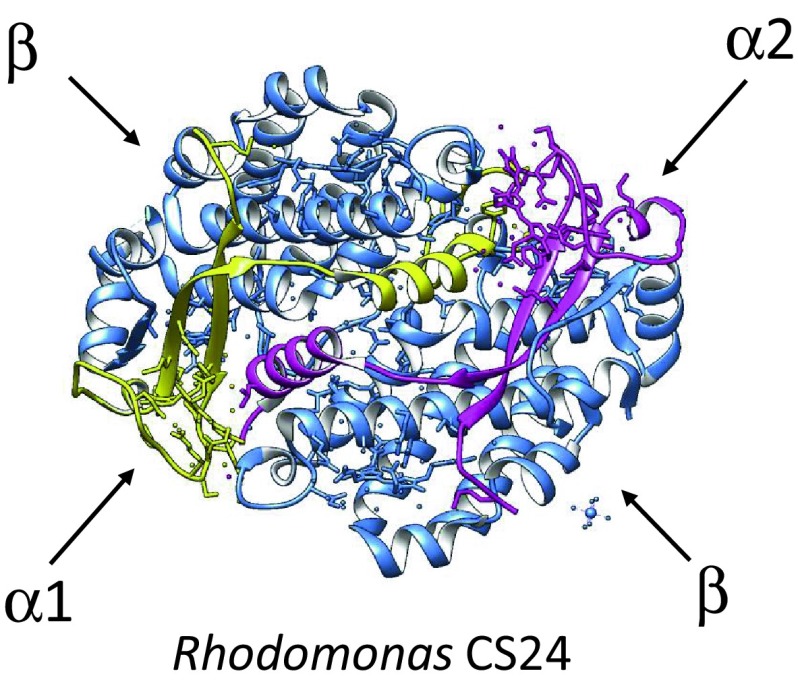
Crystal structure of *Rhodomonas* sp. PE545 (PDB 1xg0, Wilk et al. 1999). The α-1 subunit is displayed in *yellow* and the α-2 subunit in *purple*. The β-subunits are shown in *blue*

In addition to these photosynthetic organisms, several major algal groups acquired their chloroplasts by secondary endosymbiogenesis from a red algal endosymbiont (Gibbs 1981). A particularly interesting example is the cryptophyte algae, which are unique in having retained a remnant of the red algal nucleus, called the nucleomorph. The nucleomorph is located in the periplastid space, next to the chloroplast envelope, and is surrounded by two additional membranes derived from the red algal plasma membrane and the host endomembrane system (Gould et al. 2008). Cryptophytes use two different light-harvesting systems: phycobiliproteins and the chlorophyll Chl *a*/*c*-binding proteins (MacPherson and Hiller 2003; Broughton et al. 2006). The Chl *a*/*c*-binding proteins are homologous to the LHCs of the red algae and the Chl *a*/*b-*LHCs of the green lineage (Green and Durnford 1996; Durnford et al. 1999), and like these they are integrated in the thylakoid membrane. In contrast, the phycobiliprotein antenna of cryptophytes is unique (Glazer and Wedemayer 1995; MacColl et al. 1999). Rather than the complex multiprotein phycobilisome stucture, the cryptophyte phycobiliproteins are small proteins located in the thylakoid lumen (Spear-Bernstein and Miller 1989). They are compact tetrameric complexes made of two identical copies of a 18–20 kDa β-subunit (related to a phycobilisome β-subunit) and two small (8–10 kDa) subunits which were named “α-subunits,” but they have no relatedness to the phycobilisome α-subunits or to any other protein in sequence databases (Wilk et al. 1999; Doust et al. 2004). High resolution crystal structures have been determined for *Rhodomonas* sp. phycoerythrin (PE) 545 (Wilk et al. 1999) (Fig. 1) as well as several other cryptophyte phycobiliproteins (Doust et al. 2004; Harrop et al. 2014; Arpin et al. 2015).

In general, very little is known about light-harvesting mechanisms in cryptophytes. Many of these algae are adapted to low light and are suggested to take advantage of quantum coherence to improve the efficiency of energy transfer (Collini et al. 2010; Harrop et al. 2014). The photoprotective mechanism of two cryptophyte species has been studied in *Rhodomonas salina* (Kaňa et al. 2012) and *Guillardia theta* (Funk et al. 2011; Cheregi et al. 2015), and it does not appear to involve the phycobiliprotein antenna. *G. theta* has PE545, like *Rhodomonas*, and its nuclear genome has a surprisingly large number of genes encoding α-subunits (Gould et al. 2007; Curtis et al. 2012) compared to the six genes isolated by standard cloning techniques in *Rhodomonas* CS24 (Broughton et al. 2006). To find out if all the *G. theta* genes are expressed at the protein level, we used proteomics to test and optimize the gene models, and we found that all 20 α-subunit genes are indeed expressed into protein. Our data also show that the expression of the α-subunits depends on the intensity of the growth light, but there is little evidence for a specific light regulation of individual members of the α-subunit family under our experimental conditions.

## Materials and methods

### Culturing and cell counting

*Guillardia theta* cells (CCMP2712) were obtained from the Provasoli-Guillard National Center for Culture of Marine Phytoplankton. Cultures were grown in Fernbach culture flasks in h/2 media (Guillard 1975) under white light at 20 °C with light–dark cycle of 12:12 h, and shaken at 120 rpm. Light intensities were low light (7.5 µmol m^−2^ s^−1^, LL), standard light (30 µmol m^−2^ s^−1^, SL), and high light (150 µmol m^−2^ s^−1^, HL). Cultures of 1 l were started with the same number of cells (~10^5^). Every day cell number and size were determined using a calibrated Coulter Counter (Beckman Multisizer III) equipped with a 70 µm aperture. Samples were measured in triplicates. Samples were harvested after 4 days (HL), or 7 days (SL) at a cell number of 1.6 × 10^6^ cells/ml, which corresponds to the late exponential phase. Cells grown at LL were harvested after 14 days, when they had reached a density of about 1 × 10^6^ cells/ml.

### Pigment determination

Chlorophyll *a* and *c* concentrations in the cells were determined by absorption using an UV/VIS spectrophotometer (Unicam UV 550, Thermo Spectronic, UK) and calculated according to the equations of Jeffrey and Humphrey (1975). Triplicates of 5 ml of the algal suspension were filtered onto Whatman GF/F filters; the pigments were extracted by 90% acetone for 24 h at 4 °C in darkness.

### Absorption and fluorescence spectra

For absorption spectra, cells were collected on nitrocellulose membrane filters (Pragochema, Czech Republic), and the filters were then positioned in the integrating sphere of a Unicam UV550 spectrophotometer (Thermo Spectronic, UK). Absorbance was measured between 400 and 800 nm, with a bandwidth of 4 nm.

77 K fluorescence emission spectra were measured using a Fluorolog-3 spectrofluorometer (Horiba jobin Yvon, Japan). One ml of culture, diluted to the same cell number/ml (5 × 10^5^) were used in each measurement. Fluorescence was excited at 435 or 545 nm and measured from 550 to 800 nm with a bandwidth of 1 nm.

Fluorescence yield quenching (NPQ) and maximum photochemical efficiency of PSII (Fv/Fm) were measured using an AquaPen-C AP-C 100 device (Photon Systems Instruments, Czech Republic).

### Gene models and protein sequences

Gene models for 21 nuclear-encoded α-subunits were identified during the annotation of the draft genome of *G. theta* (Curtis et al. 2012). Where possible, model numbers were based on those assigned to the corresponding ESTs in Gould et al. (2008). In preparation for proteomic analysis, each gene model was carefully reexamined with respect to transcript support, intron splice sites, and model completeness, including the targeting sequences. CpeA7 and CpeA11 mapped to the same position on the genome, so CpeA7 has been deleted from the genome annotation, leaving 20 complete gene models. Alternative models were generated for several genes and, in every case, tested using the peptide sequences identified by mass spectrometry. A fasta file with the latest versions of the gene models is available at ProteomeXchange in the dataset PXD006301.

### Sequence analysis

Protein sequence alignments were generated with MAFFT version 7 (http://mafft.cbrc.jp/alignment/server/index.html) (Katoh and Standley 2013), using the default settings, and refined with BioEdit ver. 5.0.9 (Hall 1999). The analysis of the targeting sequences was performed according to Gould et al. (2008), C-terminal transit peptide cleavage sites were predicted according to Huesgen et al. (2013), and cleavage of the lumenal targeting domain by the presence of an AXA motif.

### Design of the sample set and preparation of protein extracts for mass spectrometry analysis

The sample set included five biological replicates of extracts containing soluble proteins from *G. theta* grown at LL, SL and HL. The extracts were prepared by harvesting the cells in late exponential phase: after 14 days of growth in low light, 7 days of growth in standard light, or 4 days of growth in high light. A variable volume of culture containing the same number of cells (4.5 × 10^8^) was harvested for each biological replicate and each culture condition. The pelleted cells were resuspended in 1 ml precooled breaking buffer (0.25 M Sorbitol, 20 mM HEPES, 0.4 mM Na_2_EDTA, pH ~7.5), transferred to 2 ml screw cap tubes containing 1 ml glass beads, frozen in liquid nitrogen, and kept at −80 °C until further analysis. Cells were thawed then broken on ice in darkness in a Bead beater (Glen Mills) in three cycles of 1 min with pauses of 3 min in between. Glass beads and whole cell debris were removed by centrifugation at ~600×*g* for 3 min. The green supernatant was centrifuged again at 21,000×*g* for 10–15 min to remove fragments of thylakoid membranes, giving a clear dark pink supernatant containing the phycobiliproteins as one of the major components.

The sample preparation included reduction of cysteine residues using 5 mM DTT for 20 min at 56 °C and subsequent alkylation of the thiol groups in the presence of 15 mM fresh iodoacetamide for 15 min in the dark. Next, the proteins were desalted using Zeba spin 0.5 ml columns (Thermo Fisher Scientific, Stockholm, Sweden) that were equilibrated using 50 mM Hepes pH 8.0, and the protein concentration was determined according to the method of Lowry as described by Peterson (1977). To minimize disturbance through the absorbance of the phycoerythrins, protein concentrations were measured at 750 nm.

The sample set for the preparation of in-solution digests included three technical replicates for each of the 15 biological samples. Of each sample, an aliquot containing 50 µg protein in 100 µl of 50 mM Hepes pH 8.0 was prepared, and the proteins were digested for 3 h at 37 °C in the presence of 18 ng/µl of sequencing grade trypsin (Promega Biotech AB, Nacka, Sweden). The digestion was stopped by adding 10% formic acid to a final concentration of 0.5%, and the samples were stored in −80 °C.

### Mass spectrometry analysis and bioinformatics

Automated Data Dependent Acquisition (DDA) spectra were acquired using a Synapt G2-Si mass spectrometer linked on-line to an ACQUITY UPLC M-Class System (Waters AB, Sollentuna, Sweden). The data acquisition was performed in the positive ion mode using continuum data format and lock mass calibration. In the MS mode, spectra were acquired over the range of 350–2000, and in the MS/MS mode, spectra acquisition was performed over the range of 50–2000 using charge state recognition up to four charges and eight MS/MS channels. In both the MS and MS/MS modes, a scan time of 0.4 s and an interscan scan time of 0.015 s were used. The settings for the cone voltage was 40 V. Fragmentation in the MS/MS mode was performed using MS Trap collision energy profiles ranging from 20 to 25 V in the low-mass range and from 30 to 45 V in the high-mass range. Data were acquired in the time window from 10 to 68 min.

Nanoliquid chromatography separation of peptides was performed at a flow rate of 280 nl/min and 35 °C using a combination of a Trap V/M Symmetry C18 column (100 Å, 5 µm, 180 µm × 20 mm) and HSS T3 C18 analytical column (1.8 µm, 75 µm × 250 mm) (Waters AB, Sollentuna, Sweden). The gradient was generated using 75% acetonitrile, 25% isopropanol in 0.1% formic acid (solvent B) and included the following steps: 0.5 min, 5% B; 1 min 5% B, 37 min, 41% B, 41 min, 95% B, 53 min 95% B, 57 min, 5% B. The total run time of the LC method was 69 min.

Processing of the DDA data was performed using the ProteinLynx Global Server 3.0 software (Waters AB, Sollentuna, Sweden) and the recommended settings for High Definition Data Direct Analysis (HD-DDA), including lockspray calibration and fast deisotoping in both the MS and MS/MS mode. Database searches using the peak lists of the processed mass spectra were performed using the Mascot search engine (version 2.5) in a set of databases, which included a homemade database of the gene models of the phycoerythrins of *G. theta*, a database of the JGI gene models of *G. theta* without the phycoerythrins, a database of contaminants and Glu-1-fibrinopeptide B. The search parameters permitted a mass error of 5 ppm (MS mode) and 0.05 Da, respectively (MS/MS mode) and tryptic cleavage with one missed cleavage site. Modifications included variable oxidation of methionine, and variable deamidation of asparagine and glutamine, and fixed modification of cysteine residues by carbamidomethylation. For a given database search for a biological sample, the peak-lists of the three technical replicates were merged. The Percolator of the Mascot search engine was on, and no cutoffs were applied to the percolated Mascot scores.

The use of the Percolator improved the sensitivity of the searches and allowed the detection of the unique tryptic peptides distinguishing GtcpeA1 from GtcpeA21, and GtcpeA9 from GtcpeA19. To detect N-terminal peptides, the databases searches were also performed using semitryptic cleavage, and matched spectra were inspected manually. A semiquantitative analysis of the relative expression of the α- and β-subunits was performed according to Dowle et al. (2016). The proteomics work was performed at the KBC Proteomics Core Facility at the Umeå University and the Swedish University of Agricultural Sciences, and the mass spectrometry proteomics data have been deposited to the ProteomeXchange Consortium via the PRIDE (Vizcaino et al. 2014; Vizcaíno et al. 2016) partner repository with the dataset identifiers as PXD006301 and 10.6019/PXD006301.

### Prediction of structure models

A prediction of the structures of the α- and β-phycoerythrins was performed by threading using the Phyre2 web server (Kelley et al. 2015), and the predicted structures were assessed by structural alignments to the structure templates using FATCAT (Ye and Godzik 2003, 2004a) on the public FATCAT server (Ye and Godzik 2004b; Li et al. 2006). The images of the structural alignments of the subunits of phycoerythrin 545 (PDB 1xg0) with the predicted folds of phycobiliproteins of *G. theta* were created using Chimera (Pettersen et al. 2004).

## Results and discussion

### Growth and light acclimation of *G. theta*

To investigate the growth of *G. theta* under different light intensities, cultures were grown under low light (7.5 µmol m^−2^ s^−1^, LL), standard light (30 µmol m^−2^ s^−1^, SL), and high light (150 µmol m^−2^ s^−1^, HL) in a light:dark regime of 12:12 h at 20 °C and cells counted daily. Within 4 days of culturing the high-light, resp cultures reached a cell number of 1.6 × 10^6^ cells/ml, while under standard conditions the same number of cells was reached after 7 days culturing. The low-light cells grew very slowly and after 14 days in culture had only reached ~1 × 10^6^ cells/ml. All cultures were harvested in exponential stage for pigment- and biophysical analyses.

**Fig. 2:**
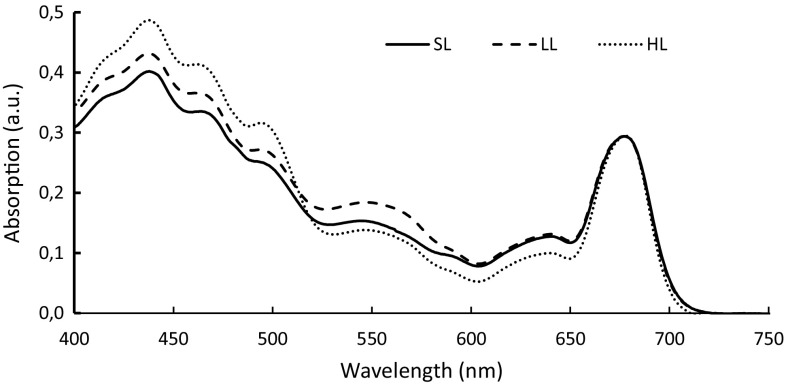
Room temperature absorption spectra of whole cells of *G. theta* grown under three light intensities: standard-light (SL) (30 µmol m^−2^ s^−1^), low-light (LL) (7.5 µmol m^−2^ s^−1^), or high-light (HL) (150 µmol m^−2^ s^−1^) conditions. Spectra were normalized at 678 nm, the maximum of PSII absorption. The same number of cells was used for each measurement. SL and HL cells were measured in late exponential phase; the slow-growing LL cells were probably in mid-exponential phase

Besides the membrane integral chlorophyll-containing antenna, *G. theta* contains phycoerythrin 545 (PE545), the red pigment–protein complex located in the thylakoid lumen (Wilk et al. 1999; Broughton et al. 2006). The pigmentation of *G. theta* cells grown at different light intensities varied noticeably: cells grown under LL conditions were deep red in color, cells grown under SL conditions had a reddish/brownish color, while cells grown under HL displayed a yellowish-green color (not shown). Absorption spectra of suspensions with similar cell density (5 × 10^5^ cells/ml) of these cultures showed absorption maxima corresponding to Chl *a* (436 and 680 nm), Chl *c* (465 nm), carotenoids (495 nm), and phycoerythrin (550 nm) (Fig. 2). The spectra were normalized at 678 nm, the maximum of PSII absorption. Absorbance at 550 nm was much higher in LL-grown cells than in HL-grown cells, reflecting their higher PE545/Chl levels as reported in earlier studies (Faust and Gantt 1973; Lichtlé 1979). Conversely, carotenoid absorption was highest in HL, resp cells (Fig. 2).

Chls *a* and *c* were estimated in acetone extracts (Table 1). Cells grown under SL had about three times the amount of Chl per cell compared to HL grown cells (Table 1), while the Chl/cell content in LL grown cells was about 60% of the one in SL cells, probably because the low level of light limited not only cell growth, but also pigment biosynthesis. The Chl *a*/Chl *c* ratio increased from 3:1 in LL, resp cells to 6:1 in HL-grown cells, indicating the Chl *c* containing antenna to be downregulated in high light. The maximal efficiency of PSII photochemistry (Fv/Fm) measured in the harvested cells demonstrated optimal PSII efficiency (higher than 0.7, the maximum reported in the literature) in all cultures, independent of the light intensity. This indicates that the cells were acclimated to their growth-light levels and are not suffering photoinhibition. This is reinforced by that fact that the non-photochemical quenching (NPQ) capacities of HL cells were similar to that of SL cells (0.59 and 0.57, respectively), while LL cells had only developed half of the protective capacity (NPQ of 0.3) (Table 1).

**Table 1:** Photosynthetic parameters of cells grown under different light intensities

	Low light (LL)	Standard light (SL)	High light (HL)
Chl/cell (pg/cell)	0.776 (±0.016)	1.300 (±0.07)	0.441 (±0.007)
Chl *a*/Chl *c*	3.56 (±0.17)	4.4 (±0.63)	6.2 (±0.3)
Fv/Fm	0.77(±0)	0.77(±0.01)	0.72 (±0.01)
NPQ	0.30 (±0.04)	0.59 (±0.03)	0.57 (±0.02)

Standard- and high-light cells were harvested in the late exponential phase at 1.6 × 10^6^ cells/ml. Low-light cells were in the mid-exponential phase when harvested at 1.0 × 10^6^ cells/ml. Average values and standard deviations are calculated from 3 to 5 biological replicates

**Fig. 3:**
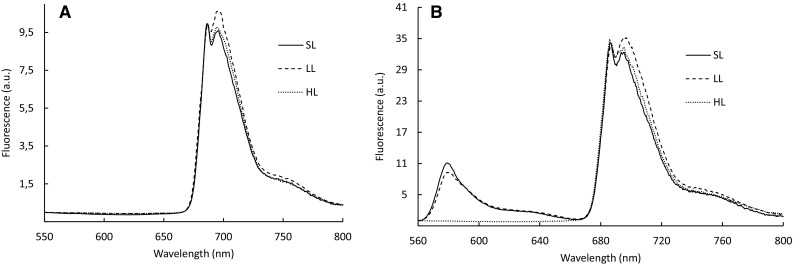
Low-temperature (77 K) fluorescence spectra of *G. theta* cells grown under standard-light (SL) (30 µmol m^−2^ s^−1^), low-light (LL) (7.5 µmol m^−2^ s^−1^) or high-light (HL) (150 µmol m^−2^ s^−1^) conditions. Chlorophyll *a* was excited at 435 nm (**a)**, and phycoerythrin PE545 was excited at 545 nm (**b**). Spectra were normalized to PSII fluorescence at 687 nm. The same number of cells was used for each measurement

Low-temperature (77 K) fluorescence emission spectra of intact cells are shown in Fig. 3. Exciting Chl *a* at 435 nm, two PSII specific emission peaks were noticed with maxima at 686 and 696 nm (Fig. 3a). The main contributor to the 695 nm maximum most likely is a low-energy chlorophyll that appears to be associated with His-114 of CP47, as in the cyanobacterium *Synechocystis* sp. PCC 6803 (Shen and Vermaas 1994). The other chlorophylls of the PSII-core complex together are represented by the 685 nm peak (Shen et al. 1993). While in cells grown at SL and HL the ratio of A686/A696 was lower than 1, in cells grown at LL the 696 nm peak was dominant. The rise of the A696 nm peak in LL cells was also seen in the 77 K spectra when PE545 was excited at 545 nm (Fig. 3b). The PE pool, which is accumulating in LL cells, seems energetically better connected to PSII. The 77 K spectra of SL- and LL cultures further displayed a third emission maxima at 578 nm, which is attributed to a pool of PE present only during the logarithmic growth phase (Cheregi et al. 2015). In HL-grown cells, this emission peak was completely absent. This pool of PE therefore either is energetically more strongly coupled to the photosystems during HL or it is degraded.

### Gene models and expression of the α-subunits

**Fig. 4:**
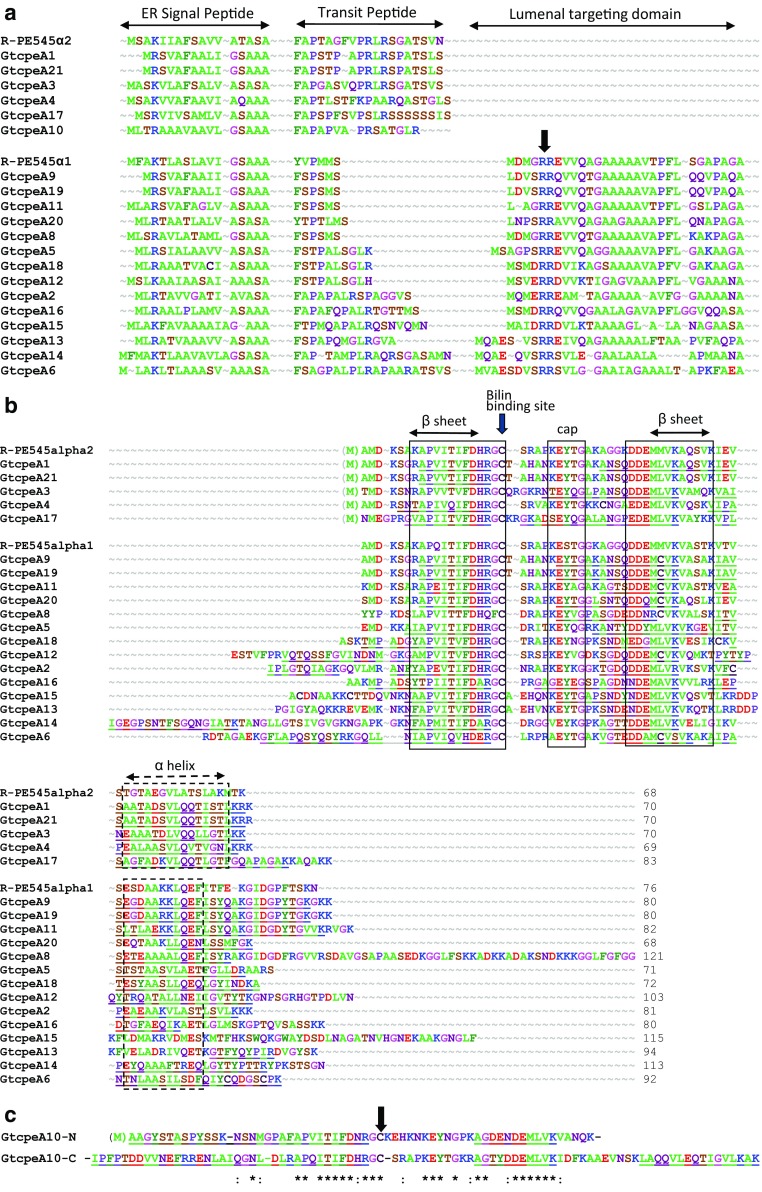
Multiple sequence alignment of the *G. theta* α-subunit sequences colored according to chemical similarity. **a** Precursor targeting sequences. All precursors start with an ER signal peptide, cleaved from the following chloroplast transit peptide at the conserved motif AXA_(F/Y) (Gruber et al. 2007). The C-terminal cleavage site of the chloroplast transit peptide is predicted according to Huesgen et al. (2013) with a conserved Leu or Met at the -2 position. Where present, the lumenal targeting domain has a twin arginine motif near the N-terminus (*arrow*) and a hydrophobic core, typical of proteins targeted to the thylakoid lumen via the TAT import machinery, and a C-terminal AXA cleavage site to release the mature protein. **b** Mature proteins aligned with the α1- and α2-sequences from the crystal structure of PE545 from *Rhodomonas* sp. CS24 (Wilk et al. 1999). Peptides identified by mass spectrometry are underlined. N-terminal Met are in parenthesis to indicate the high probability that they are removed by Met aminopeptidase (Huesgen et al. 2013; reviewed by Giglione and Meinnel 2001). Highly conserved regions corresponding to secondary structural elements of the *Rhodomonas* structure are boxed. The EYxG motif forms a cap on the end of one of the β-subunit helices. *Arrow*, bilin-binding site. **c** Internal duplication of GtcpeA10. The mature sequence was split into N- and C-terminal halves and aligned to show sequence relatedness. Both halves a bilin-binding site. Peptides identified by mass spectrometry are *underlined. Parenthesis*, removed N-terminal Met; *arrow*, bilin-binding site

The nuclear genome of *G. theta* contains 20 different genes encoding PE545 α-subunits, annotated *GtcpeA1-6* and *GtcpeA8-21* (Curtis et al. 2012). In our reexamination of the gene models, the incomplete *GtcpeA7* model mapped to the same position on the genome as *GtcpeA11*, and it was therefore deleted from the genome annotation. The 20 genes are scattered across a number of scaffolds, with the exception of two pairs of neighboring genes: *GtcpeA1-A9* on scaffold 57 and *GtcpeA21-A19* on scaffold 117. *GtcpeA9* and *GtcpeA19* encode almost identical protein sequences, as do *GtcpeA1* and *GtcpeA21*. These closely related pairs are clearly the result of a duplication of the whole gene pair followed by transposition. Like the three pairs of α-subunit genes in *Rhodomonas sp*. CS24 (Broughton et al. 2006), the two genes are divergently transcribed. One member of each pair encodes a slightly longer protein precursor with the full tripartite targeting sequence including the lumenal TAT targeting domain (α1-type), whereas the other has only the ER signal peptide plus the transit peptide (α2-type) (Fig. 4). Since the plastid-encoded β-subunit also lacks the lumenal targeting domain (LTD), this suggested that the tetramer is assembled in the plastid stroma and then targeted across the thylakoid membrane by the α1-type protein (Broughton et al. 2006; Gould et al. 2008). The *G. theta* α-subunit sequences are closely related to those of *Rhodomonas*, so it is expected that the holoprotein will have the same three-dimensional structure as the PE545 of *Rhodomonas sp*. CS24 (Wilk et al. 1999).

The rest of the α-subunit genes do not fall neatly into pairs with and without LTDs (Fig. 4a). There are 14 genes encoding a complete tripartite targeting sequence that includes an LTD, and 6 that have no LTD. They all have a canonical endoplasmic reticulum signal peptide (SP), with predicted SP-transit peptide cleavage site AXA-FXP as determined for diatoms (Gruber et al. 2007; Huesgen et al. 2013). The transit peptides are varied in length but have the same overall composition as diatom and plant transit peptides, being enriched in hydroxylated amino acids (S and T) and with at least one positive charge except for the shortest ones. C-terminal cleavage sites were predicted according to Huesgen et al. (2013), with a conserved Leu or Met at the -2 position. All LTDs have the conserved twin arginine motif followed by a hydrophobic domain characteristic of the TAT-transport system and an AXA-motif at the C-terminal cleavage site, with the exception of *GtcpeA13* which most likely has the C-terminal cleavage site VFA.

In Fig. 4b, the predicted mature protein sequences are aligned with the α1- and α2-sequences from the crystal structure of *Rhodomonas* PE545 (Wilk et al. 1999; Harrop et al. 2014). All sequences share two blocks of high sequence similarity, which correspond to the two β-strands seen in the crystal structure, and the conserved FDxRGC motif, where the Cys residue is covalently linked to dihydrobiliverdin. They also share a somewhat conserved block predicted to form the characteristic C-terminal alpha helix. These structural elements are so conserved across all six cryptophyte phycobiliproteins structures, which have been determined at high resolution (Harrop et al. 2014; Arpin et al. 2015), that it is safe to conclude that the *G. theta* α-subunits will also have a similar fold when assembled with their β-subunit partners.

A number of the sequences encoded by genes with LTDs have long N- and/or C-terminal extensions. The N-terminal extensions of GtcpeA2 and GtcpeA14 are supported by peptide sequences (Fig. 4b, underlined), and probably represent the true mature N-termini. There is also peptide support for the extensions of GtcpeA6 and GtcpeA12. At the C-terminal end, the distinction between α1 type (with LTD) and the α2 type (shorter, no LTD) breaks down, with both long and short C-termini, many with peptide support as detailed in the next section about the proteomics analysis.

A most interesting gene model is GtcpeA10, which has a second FDxRGC motif as the result of a partial internal duplication. Figure 4c shows that the N- and C-terminal parts of this gene model have a higher sequence similarity to one another than to the other α-subunits of this group. Both parts have high peptide coverage. We have found homologs to this sequence in transcriptomes of two other cryptophytes with PE545 and one with PE566 (data not shown) showing that this duplicated sequence arose in the common ancestor of the clades with PE and has not been eliminated by selection or degenerated into a pseudogene. This implies that this gene model represents a functional protein, but it raises the question of how it would interact with the other α- and β-subunits.

### Proteomics analysis

To test the gene models and their expression, our proteomic analysis included *G. theta* cultures grown under white light of three different intensities: 30 µmol m^−2^ s^−1^ (SL), 7.5 µmols m^−2^ s^−1^ (LL), and 150 µmols m^−2^ s^−1^ (HL). For each light condition, five biological replicates were grown, of which in turn three technical replicates were analyzed by mass spectrometry. In total, our dataset consists of 45 LC-MS/MS runs and the processed data contain 69615 spectra, of which 5950 match tryptic peptides of the phycobiliproteins. The datasets from the LC-MS/MS analysis were first used for database searches with the Mascot search engine, to verify and optimize the gene models. Table 2 gives the percolated Mascot scores, which have higher sensitivity than standard Mascot scores [for explanation. see, e.g., (Brosch et al. 2009)]. Table 2 shows that in the samples from algae that were grown under SL and LL conditions, all 20 α-subunits were detected. For most of the α-subunits, the identified peptides have two or more unique sequences (Table 3), and together they cover large parts of the predicted mature proteins, which are underlined in Fig. 4b, c.

**Table 2:** Phycoerythrins of *G. theta* expressed under different light growth conditions

Gene model	Low-light growth conditions					Standard-light growth conditions					High-light growth conditions				
	1	2	3	4	5	1	2	3	4	5	1	2	3	4	5
GtcpeA1	988	1298	478	700	697	1421	667	730	1390	860	971	733	495	802	855
GtcpeA2	102	130	106	47	42	204	181	68	280	276			26		
GtcpeA3	511	453	254	274	369	983	423	423	669	394	86	453	172	313	183
GtcpeA4	208	256	83	67	116	387	226	172	205	89					
GtcpeA5	138	223	169	138	184	466	223	297	378	285		64	62	176	48
GtcpeA6	342	673	299	563	379	653	256	439	753	810	332	423	216	581	377
GtcpeA8	158	270	136	18	200	327	262	239	368	365		42			
GtcpeA9		909			381										
GtcpeA10	858	1037	384	537	539	1421	621	554	1553	876	81	509	194	220	178
GtcpeA11	365	411	134	236	191	612	324	223	716	535	240	323	202	302	244
GtcpeA12	576	825	325	500	301	613	298	425	728	426	133	412	237	560	583
GtcpeA13	31	89			25	172	102	93	92	93		50	35	42	18
GtcpeA14	381	440	213	332	290	750	283	292	812	646	162	189	224	367	152
GtcpeA15	355	477	205	333	281	484	210	332	581	441	231	194	130	278	318
GtcpeA16	774	1679	810	700	1019	1913	779	793	1461	996	1026	925	889	1592	909
GtcpeA17	78	359	165	98	159	567	479	217	253	147					
GtcpeA18	516	968	381	589	610	1448	953	440	529	507	263	301	460	811	438
GtcpeA19		975	269		404			370	1136	852	404	375	278		
GtcpeA9 or GtcpeA19				323*		754*								477*	
GtcpeA20	543	1031	375	675	615	1282	573	605	1514	900	638	447	405	558	608
GtcpeA21	973	1241	469	681	677	1283	660	702	1320	816	883	730	463	767	848
Gt-Beta	2308	3619	2077	2400	1500	2869	1151	1437	4407	4133	1443	1304	1139	1768	1581

Percolated Mascot scores of the antenna gene models identified in fifteen biological replicates

The database searches were performed with the Mascot 2.5 search engine, and the search results have ≥95% confidence and a false discovery rate of ≤1%. Each search includes the peak list files of three technical replicates, and the table summarizes the results from 45 LC-MS/MS runs. If a search did not give a significant score for a gene model, the cell is empty

*Identified peptides could not be destinguished between GtcpeA9 and GtcpeA19 in database search

**Table 3:** Experimentally detected unique peptides of the phycobiliproteins of *G. theta*

Protein	Start and end in precursor	Start and end in predicted mature protein	Unique tryptic peptide	Highest peptide score	Lowest peptide expectation value	Spectra with expectation values <0.05
Beta		1–7	MLDAFSR (N-terminus)	47.64	1.70E−05	14
		16–28	AAYVGGADLQALK	117.63	1.70E−12	90
		16–29	AAYVGGADLQALKK	90.98	8.10E−10	147
		85–91	DGEIILR	39.35	0.00012	6
		92–108	YVSYALLSGDSSVLEDR	155.02	3.20E−16	289
		115–129	ETYSSLGVPANSNAR	120	1.00E−12	230
		130–149	AVSIMKACAVAFINNTASQR	145.61	2.80E−15	30
		136–149	ACAVAFINNTASQR	155.02	3.20E−16	160
		150–171	KLSTPQGDCSGLASECASYFDK	114.82	3.30E−12	31
		151–171	LSTPQGDCSGLASECASYFDK	155.02	3.20E−16	26
GtcpeA1	42–51	9–18	APVITVFDHR	73.05	5.00E−08	181
GtcpeA2	59–68	1–10	IPLGTQIAGK (N-terminus)	25.67	0.0027	30
	75–88	17–30	ANFYAPEVTIFDHR	76.29	2.40E−08	6
	102–112	44–54	TGDQDDEMLVR	89.35	1.20E−09	10
	118–127	60–69	VFCPEAEAAK	13.82	0.041	1
	128–137	70–79	VLASTLSVLK	77.34	1.80E−08	39
GtcpeA3	45–54	9–19	APVVTVFDHR	91.3	7.40E−10	35
	62–80	26–44	NTEYQGLPANSQDDEMLVK	155.02	3.20E−16	90
	61–80	25–44	RNTEYQGLPANSQDDEMLVK	18.77	0.013	1
	86–105	50–69	VAINEAAATDLVQQLLGTLK	120.64	8.60E−13	16
	86–106	50–70	VAINEAAATDLVQQLLGTLKK (C-terminus)	106.65	2.20E−11	13
GtcpeA4	43–55	6–18	SNTAPIVQIFDHR	92.01	6.30E−10	35
	84–104	47–67	VIPAPEALAASVLQVTVGNLK	93.35	4.60E−10	12
GtcpeA5	63–74	6–17	AIAPVITIFDHR	112.95	5.10E−12	20
	88–98	31–41	ANTYDDYMLVK	58.6	1.40E−06	16
	87–98	30–41	KANTYDDYMLVK	58.06	1.60E−06	8
	101–124	44–52	GEVITVSTSTAASVLAETFGLLDR	37.6	0.00017	1
	99–124	42–67	VKGEVITVSTSTAASVLAETFGLLDR	141.73	6.70E−15	32
GtcpeA6	78–89	9–20	GFLAPQSYQSYR	91.23	7.50E−10	120
	90–106	21–37	KGQLLNIAPVIQVHDER	55.2	3.00E−06	16
	91–106	22–37	GQLLNIAPVIQVHDER	97.83	1.60E−10	16
	120–132	51–63	VGTEDDAMCVSVK	132.58	5.50E−14	174
	135–161	66–92	AIPANTNLAASILSDFQIYCQDGSCPK (C-terminus)	126.81	2.10E−13	129
GtcpeA8	77–90	25–38	EYVGPASGDEDDNR	76.52	2.20E−08	6
	99–117	47–65	ITVSETEAAAALQEFISYR	154.66	3.40E−16	41
GtcpeA9	98–107	48–57	IAVSEGDAAK	26.6	0.0022	3
GtcpeA10	34–46	2–14	AAGYSTASPYSSK (N-terminus)	28.85	0.0013	3
	47–64	15–32	NSNMGPAFAPVITIFDNR	155.02	3.20E−16	71
	79–89	47–57	AGDENDEMLVK	28.24	0.0015	10
	95–107	63–75	IPFPTDDVVNEFR	145.05	3.10E−15	50
	95–108	63–76	IPFPTDDVVNEFRR	25.22	0.003	7
	109–120	77–88	ENLAIQGNLDLR	67.25	1.90E−07	18
	109–130	77–98	ENLAIQGNLDLRAPQITIFDHR	26.91	0.002	3
	121–130	89–98	APQITIFDHR	125.77	2.60E−13	79
	144–154	112–122	AGTYDDEMLVK	32.63	0.00055	29
	166–179	134–147	LAQQVLEQTIGVLK	84.23	3.80E−09	29
	166–181	134–149	LAQQVLEQTIGVLKAK (C-terminus)	31.42	0.00072	1
GtcpeA11	59–68	8–17	APEITIFDHR	89.96	1.00E−09	61
	82–92	31–41	AGTSDDEMCVK	42.6	5.50E−05	10
	98–107	47–56	VEASLTLAEK	27.29	0.0019	25
	119–129	68–78	GIDGDYTGVVK	70.2	9.50E−08	55
GtcpeA12	64–79	8–23	VQTQSSFGVINDNMGK	129.58	1.10E−13	51
	80–91	24–35	GAMPVITVFDHR	67.79	1.70E−07	14
	105–115	49–59	SGDQDDEMCVK	32.64	0.00054	6
	120–129	64–73	TPYTYPQYTR	52.09	6.20E−06	52
	130–145	74–89	QATALLNEIIGVTYTK	155.02	3.20E−16	50
GtcpeA13	79–90	18–29	NFAPVITVFDHR	66.65	2.20E−07	11
	105–115	44–54	SNDENDEMLVK	18.13	0.015	2
	141–149	80–88	GTFYQYPIR	64.48	3.60E−07	32
GtcpeA14	67–84	1–18	IGEGPSNTFSGQNGIATK (N-terminus)	131.28	7.50E−14	65
	85–99	19–33	TANGLLGTSIVGVGK	146.89	2.00E−15	46
	107–118	41–52	NFAPMITIFDAR	95.41	2.90E−10	43
	132–142	66–76	AGTTDDEMLVK	28.18	0.0015	1
	150–160	84–94	VPEYQAAAFTR	73.4	4.60E−08	51
	161–171	95–105	EQLGYTYPTTR	57.5	1.80E−06	55
GtcpeA15	78–89	17–28	NAAPVITIFDHR	96.09	2.50E−10	55
	98–114	37–53	EYTGAPSNDYNDEMLVK	155.02	3.20E−16	60
GtcpeA16	66–81	4–19	MPADSYTPIITIFDAR	155.02	3.20E−16	96
	86–105	24–43	GAGEYEGSPAGDNNDEMAVK	155.81	2.60E−16	186
	111–122	49–60	LEPDTGFAEQIK	57.2	1.90E−06	7
	110–122	48–60	KLEPDTGFAEQIK	88.18	1.50E−09	31
	111–131	49–69	LEPDTGFAEQIKAETLGLMSK	19.97	0.01	1
GtcpeA17	44–55	8–19	GVAPIITVFDHR	44.81	3.30E−05	3
	60–81	24–45	GKADSEYQGALANGPEDEMLVK	28.48	0.0014	3
	62–81	26–45	ADSEYQGALANGPEDEMLVK	154.68	3.40E−16	31
	87–96	51–60	VPLSAGFADK	35.26	0.0003	3
	97–113	61–77	VLQQTLGTFGQAPAGAK	155.02	3.20E−16	21
GtcpeA18	59–75	4–20	TMPADGYAPVITVFDHR	113.61	4.40E−12	81
	89–99	34–44	SNDMEDGMLVK	54.99	3.20E−06	54
	107–126	52–71	VTESYAASLLQEQLGYINDK	153.61	4.40E−16	60
	107–127	52–72	VTESYAASLLQEQLGYINDKA (C-terminus)	154.66	3.40E−16	64
GtcpeA19	98–107	48–57	IAVSEGDAAR	51.02	7.90E−06	15
GtcpeA20	77–93	25–41	EYTGGLSNTQDDQMCVK	155.02	3.20E−16	84
	109–120	57–68	LLQENLSSMFGK (C-terminus)	153.03	5.00E−16	56
GtcpeA21	42–51	9–18	APVVTIFDHR	66.35	2.30E−07	176

The table shows the tryptic peptides with unique sequences of the phycobiliproteins of *G. theta* that are detected in the Mascot database searches. The search parameters permitted a mass error of 5 ppm and one missed cleavage site. The expectation value corresponds to the E-value of a Blast search result, and expectation values <0.05 have a confidence of >95%

For GtcpeA8 and GtcpeA15, the sequence coverage was lower, but for each of these proteins, two unique peptides were identified, which provides sufficient confidence for their identification. In the samples of algae that were grown under HL conditions, GtcpeA4 and GtcpeA17 were not detectable, which is probably due to their amount being below the detection threshold of our assay. In addition, GtcpeA8 was barely detectable under HL conditions.

The sequences of the GtcpeA1 and GtcpeA21 precursor proteins differ by only three amino acid residues, and those of GtcpeA9 and GtcpeA19 by only two (Fig. 4). The mature proteins of GtcpeA1 and GtcpeA21 are distinguished from each other by only one tryptic peptide, which is APVITVFDHR in GtcpeA1 and APVVTIFDHR in GtcpeA21. However, both peptides were detected by more than 100 spectra (Table 3) and in all of the 15 biological samples analyzed (Table 2), which supports the expression of these proteins. GtcpeA9 has the unique tryptic peptide IAVSEGDAAK and GtcpeA19 the unique tryptic peptide IAVSEGDAAR, neither of which is found in any other protein. The peptide IAVSEGDAAR of GtcpeA19 was detected in 9 of the 15 biological samples analyzed, and the peptide IAVSEGDAAK of GtcpeA9 in two samples, which supports the expression of these proteins. In summary, our data support the expression of all 20 α-subunit proteins, and they are also consistent with the presence of 225 EST sequences for the α-subunit proteins in Genbank, which include at least one high-quality EST sequence for each α-subunit.

The experimentally identified peptides also allow an assignment of some of the N- and C-terminal sites of the α1-type (with LTD) and α2-type (no LTD) α-subunits. The long N-terminal extensions of the α1-type GtcpeA2 and GtcpeA14 are supported by the peptides IPLGTQIAGK and IGEGPSNTFSGQNGIATK (Table 3; Fig. 4b, underlined), which probably represent the mature N-termini of these proteins. At the C-terminal end, the peptides AIPANTNLAASILSDFQIYCQDGSCPK and VTESYAASLLQEQLGYINDKA provide support for the predicted C-termini of the gene models of GtcpeA6 and GtcpeA18, and the peptide LLQENLSSMFGK for the one of GtcpeA20. For many of the other α1-subunits, the support of the C-terminal parts of their gene models through identified peptides is good, although no peptides were found to support the very long GtcpeA8 and GtcpeA15 tails. Since the long 3′-ends of these genes have not been experimentally verified with 3′-RACE, it is possible that they are the result of sequencing errors.

As for the α2-type GtcpeA10, the peptide AAGYSTASPYSSK matches the predicted N-terminus. The N-terminal Met of the putative mature GtcpeA10, which precedes the sequence AAGYSTASPYSSK, is likely cleaved off after the import into the chloroplast stroma (Huesgen et al. 2013). In addition, the peptide LAQQVLEQTIGVLKAK supports the C-terminal end of this protein. Finally, there is also support for the C-terminal end of GtcpeA3 by the peptide VAINEAAATDLVQQLLGTLKK. As for the other α2-type α-subunits, no peptides that match the predicted C-termini of their gene models were detected, but the coverage of their C-terminal parts is good (Fig. 4b, c).

The low level or the absence of peptides for some proteins under HL (Table 2) suggested that some subunits might have a control function that is regulated by the intensity of the growth light. Due to ion suppression in electro spray-ionization, quantization by direct comparison of peptides from different proteins is not possible. Usually, relative quantization methods are applied, in which the relative levels of individual proteins under different conditions are measured. In this study, we used a semiquantitative evaluation by peptide counting to test if individual α-subunits are regulated by the different intensities of the white growth light, under which the algae were grown. The goal of this approach was to reject the working hypothesis that no individual α-subunit is regulated by light under the experimental conditions of this study.

**Fig. 5:**
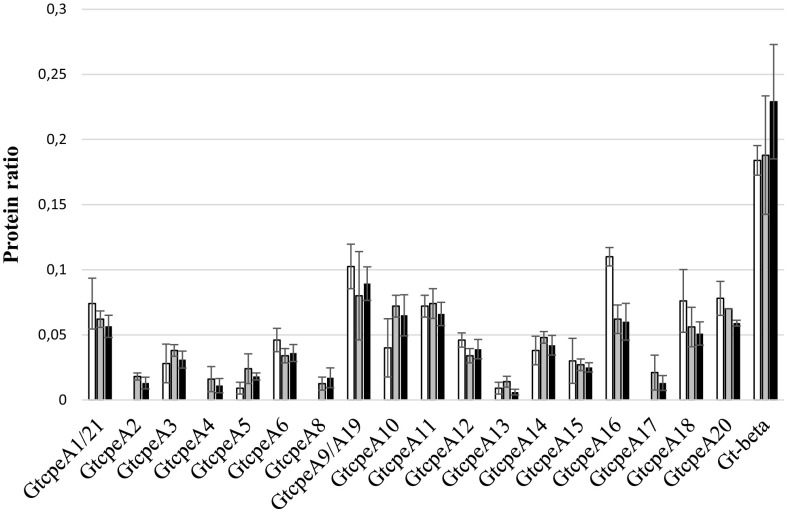
Relative expression of the individual phycobiliproteins of *G. theta* relative to the pool of all phycobiliproteins. *G. theta* cells were grown either at low light (LL, *black bars*), standard light (SL, *gray bars*), or high light (HL, *white bars*). For each light condition, the ratios of the individual phycobiliproteins to the entire phycobiliprotein pool were determined by semiquantitative peptide counting according to Dowle et al. (2016). GtcpeA1 and GtcpeA21, and GtcpeA9 and GtcpeA19 were quantified as groups and not as individual proteins, because of their high sequence similarity. The data are based on five biological replicates with three technical replicates each. The standard deviations were calculated from the ratios of the phycobiliproteins obtained for each biological replicate and included in the graph as *error bars*

Our electrospray ionization mass spectrometry method allows no absolute quantization but only the relative comparison of unique peptides of the individual phycobiliproteins (e.g., the presence of GtcpeA14 at different light intensities). Figure 5 shows for each light condition the relative ratios of the individual α- and β-subunits to the entire pool of the phycobiliprotein peptides. The result of this evaluation is that the relative ratios of each α- and β-subunit do not change significantly under LL, SL or HL conditions within the limits of the standard deviations of our quantization. There is one outlier for GtcpeA16 under HL conditions, but it is not strong enough to reject the hypothesis that the relative ratios of the α- and β-subunits do not change significantly, when the algae are grown under white light of different intensities. It therefore appears that the PE545 antennas function as a pool that is regulated by light intensity. As long as the spectral composition of the growth light does not change, there seems to be no need for the algae to change the composition of the subunit pool, and it is enough if the algae adjust the pool size to adapt to the light intensity in the surrounding environment. Studies of subunit expression under different growth conditions and a more accurate quantization than our peptide counting experiment might reveal more details, which might change this picture, but that is beyond the scope of this study, and at this point, there is no evidence for the differential light regulation of the functions of individual α- and β-subunits under white growth light.

### Structure prediction

**Fig. 6:**
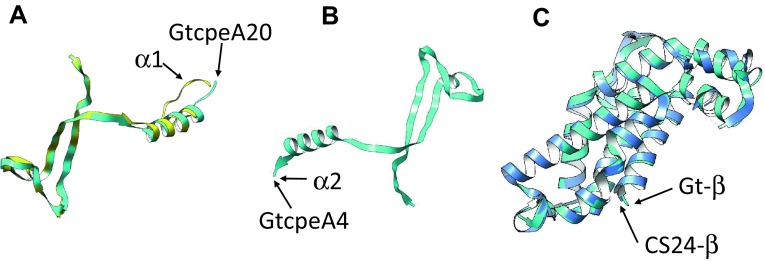
Structure predictions of the α-subunits. The folds of GtcpeA4, GtcpeA20, and the β-subunit of *G. theta* PE545 were predicted using Phyre 2 (Kelley et al. 2015) and aligned using FATCAT (Ye and Godzik 2003) to the experimental folds of their models in the PE545 of *Rhodomonas* sp. CS24 (Doust et al. 2004). **a** Alignment of GtcpeA20 (*cyan*) to the α1-subunit (*yellow*) of the *Rhodomonas* PE545 tetramer (Fig. 1). The alignment has sequence coverage of 99% and a confidence of 100%. **b** Alignment of GtcpeA4 (*cyan*) to the α2-subunit (*yellow*) of the *Rhodomonas* PE545 tetramer. The alignment has sequence coverage of 99% and a confidence of 100%. The similarity between these folds is so high that the color of the *Rhodomonas* chain is not visible. **c**. Alignment of the *G. theta* β-subunit (Gt-β, *cyan*) to that of *Rhodomonas* PE545 (CS24-β, *blue*). The alignment has sequence coverage of 100% and a confidence of 100%

To address the question of whether the gene models of the α- and β-subunits of *G. theta* PE545 are likely to have the same fold as the subunits of PE545 of *Rhodomonas* sp. CS24 (Doust et al. 2004) and related antenna complexes, we searched for suitable fold models using the Phyre 2 server (Kelley et al. 2015) (Fig. 6). We found that all sequences of the mature α-subunits aligned with 99.9% confidence or higher to the folds of the α-1 and α-2 subunits of *Rhodomonas* sp. CS24 (d1xg0a_ and d1xg0b_) (Doust et al. 2004) or to the fold of the α-1 subunit of phycocyanin PC645 of *Chroomonas* sp. CCMP270 (c4lmsA_) (Harrop et al. 2014). Both folds are very similar and can be aligned to one another with high confidence, with the exception of the N-terminal extensions and C-terminal tails. This was not surprising, since Harrop et al. (2014) showed that the three-dimensional structures of four different cryptophyte phycobiliproteins (PE545, PC645, PC612, and PE555) could be superimposed. A critical assessment of these alignments shows, however, that 13 of the 20 α-subunits of *G. theta* are predicted to have more than 50% disorder, which means that their structure predictions have low confidence. Nevertheless, good alignments with a sequence coverage of 84–99% were obtained for GtcpeA4, GtcpeA5, GtcpeA11, GtcpeA16 and GtcpeA20. As an example, the alignments of GtcpeA4 and GtcpeA20 to the fold of the corresponding *Rhodomonas* sp. CS24 α-subunits are shown in Fig. 6a, b. The central parts of GtcpeA13 and GtcpeA15 also give good alignments with a sequence coverage of 69 and 57%, but their N- and C-terminal tails do not match the model fold. Searches for structure models for the β-phycoerythrin of *G. theta* resulted in an excellent match to the β-phycoerythrin of *Rhodomonas* sp. CS24 (Doust et al. 2004) (Fig. 6c), which is expected since their primary sequences are almost identical. In summary, our modeling work suggests that the phycobiliprotein complexes of *G. theta* have similar three-dimensional structures as that of *Rhodomonas sp*. CS24, even though some differences in the N- and C-terminal extensions of some α-subunits might exist.

The expressions of 6 different α1-type and 14 different α2-type proteins raise the intriguing possibility that the *G. theta* chloroplast has multiple types of tetramers with different combinations of α- and β-subunits. In support of this, the pioneering work of Hiller and Martin (1987) on purified PE545 from *Rhodomonas* sp. CS24 (then called *Chroomonas*) resolved four fractions with distinctive isoelectric points using a chromatofocusing column. The major fraction (pI 6.24) contained both α1- and α2-type bands, while a minor fraction (pI 7.16) contained only the lighter α2-type, and the other two fractions only the heavier α1-type. This suggested the existence of α_1_β α_1_β and α_2_β α_2_β as well as α_1_β α_2_β tetramers. In the case of *G. theta*, there could be an even greater variety of tetramers.

This brings up another complication. In light of our current knowledge of targeting to secondary plastids: the α_2_β α_2_β tetramers could not be transported into the thylakoid lumen because neither α-subunit would have a LTD. However, if they escaped degradation by stromal proteases, there is no reason why they could not associate with the stromal surface of the thylakoid membrane and transfer energy to the photosystems, since there appears to be no preferred orientation or packing arrangement for the tetramers within the lumen. It would then be possible to envisage redox or other control being exerted on at least a small fraction of the phycobiliprotein population.

## Conclusions

Our proteomics analysis shows that all the 20 genome-predicted α-subunits of *G. theta* PE545 are expressed at the protein level, and suggests that the PE545 α-subunits operate as a pool that is regulated up and down depending on the light intensity. The similarity of these sequences to those of the published crystal structure of *Rhodomonas* sp. CS24, as well as the very high similarity of the β-subunit sequences in these two species suggest that all the PE545s have similar three-dimensional structures. GtcpeA10 with its internal duplication might be able to link two partial tetramers or to form oligomers.
